# Generation of Immortalised But Unstable Cells after hTERT Introduction in Telomere-Compromised and p53-Deficient vHMECs

**DOI:** 10.3390/ijms19072078

**Published:** 2018-07-17

**Authors:** Aina Bernal, Elisenda Zafon, Daniel Domínguez, Enric Bertran, Laura Tusell

**Affiliations:** Unitat de Biologia Cel·lular, Facultat de Biociències, Universitat Autònoma de Barcelona, 08193 Cerdanyola del Vallès, Spain; aina.bernal@uab.cat (A.B.); elisenda94@gmail.com (E.Z.); irgu00@gmail.com (D.D.); enbegadol@gmail.com (E.B.)

**Keywords:** human mammary epithelial cells, telomere dysfunction, chromosome instability, p53, hTERT, cancer

## Abstract

Telomeres, the natural ends of chromosomes, hide the linear telomeric DNA from constitutive exposure to the DNA damage response with a lariat structure or t-loop. Progressive telomere shortening associated with DNA replication in the absence of a compensatory mechanism culminates in t-loop collapse and unmasked telomeres. Dysfunctional telomeres can suppress cancer development by engaging replicative senescence or apoptosis, but they can also promote tumour initiation when cell cycle checkpoints are disabled. In this setting, telomere dysfunction promotes increasing chromosome instability (CIN) through breakage-fusion-bridge cycles. Excessive instability may hamper cell proliferation but might allow for the appearance of some rare advantageous mutations that could be selected and ultimately favour neoplastic progression. With the aim of generating pre-malignant immortalised cells, we ectopically expressed telomerase in telomere-compromised variant human mammary epithelial cells (vHMECs), proficient and deficient for p53, and analysed structural and numerical chromosomal aberrations as well as abnormal nuclear morphologies. Importantly, this study provides evidence that while immortalisation of vHMECs at early stages results in an almost stable karyotype, a transient telomere-dependent CIN period—aggravated by p53 deficiency—and followed by hTERT overexpression serves as a mechanism for the generation of immortal unstable cells which, due to their evolving karyotype, could attain additional promoting properties permissive to malignancy.

## 1. Introduction

Telomerase reactivation is a hallmark of carcinogenesis, and the vast majority of human tumours have telomerase activity by upregulating expression of telomerase’s catalytic subunit (hTERT) [1]. In addition to replicative immortality, cancer cell hallmarks include sustained proliferative signalling, inhibition of growth suppressors and resistance to cell death [2]. Underlying these hallmarks is the presence of chromosome instability (CIN), a process that fuels genetic heterogeneity among a cell population. It is thought that the presence of an unstable genome expedites the acquisition of traits enabling malignity [2,3,4], though it has also been proposed that it is a simple by-product of tumour evolution [5].

Multiple mechanisms have been described to enable the development of CIN [6,7,8,9]. Among them, telomere damage is believed to trigger CIN when critically short telomeres become dysfunctional and prone to chromosomal fusions in cells lacking proper cell cycle checkpoints. In human tissues, progressive telomere shortening occurs due to the inability of polymerases to fully replicate the chromosome ends [10,11]. Excessive reduction of the telomere length renders telomeres dysfunctional and the onset of replicative senescence [12,13]. Indeed, human fibroblasts accumulate spontaneous telomere-dysfunction induced foci (TIFs) during cellular lifespan [14]. Cells keep dividing until they reach 4–5 TIFs, and above this threshold, persistent telomere damage enforces cells to become senescent through p53-dependent signalling [14,15,16]. Notably, cells with abrogated checkpoints may escape the growth arrest and be more tolerant of rampant CIN when fully deprotected telomeres become fusogenic. If left unchecked, this instability will eventually reach lethal levels in the transforming cells, thereby presenting crisis, a second block to the development of cancer [17,18]. It is currently believed that full malignant progression arise from cells in which telomerase or Alternative Lengthening of Telomeres (ALT)-pathway activation and restoration of telomere function appears after a period of telomere instability [18]. At least in murine models, telomerase reactivation in the setting of a pre-existing telomere-induced genome instability is an active driver of carcinogenesis [19].

Previous telomere and cytogenetic studies have documented CIN, reduced telomere length and telomere end fusions in early-stage human breast cancers [20,21,22,23], thus supporting telomere dysfunction as a driver of CIN and an inducer of intratumour diversity in this emerging malignancy [24]. These findings, along with the detection of telomerase activity in some breast carcinomas in situ [25,26,27,28], suggest that immortalisation of telomere unstable cells through the activation of telomerase could be an early event in the progression of breast carcinogenesis.

Here, with the aim of exploring the impact of hTERT overexpression in breast epithelial cells displaying short dysfunctional telomeres, we have taken advantage of human mammary epithelial cells (HMEC), which have been determined to mimic breast carcinogenesis in vitro [20]. Remarkably, HMECs acquire an extended lifespan in vitro due to the absence of p16^INK4a^ expression [29]. In these proliferating variant HMECs (vHMEC), progressive telomere shortening results in the transit of telomeres from a closed state to an uncapped state and, ultimately, to the gradual appearance of fully unprotected telomeres that are continuously repaired by fusing with each other [30,31]. This reduces the initial damage and allows massive remodelling and scrambling of the genome through endless breakage-fusion-bridge (BFB) cycles on proliferating cells (reviewed in [32]). Nevertheless, these massively reorganised cells ultimately succumb to p53-dependent agonescence, or crisis if p53 function is abrogated [33]. Our studies establish that hTERT overexpression in vHMEC cells before CIN is unleashed enables the immortalisation of cells with a relatively stable karyotype. By contrast, immortalisation of cells after a brief episode of chromosomal instability offered by dysfunctional telomeres avoids the persistent mutator phenotype that hampers cell proliferation. Beyond restoring genome stability and eliminating the DNA-damage signals of unprotected telomeres, the provided data demonstrates the presence of a still-evolving karyotype due to persistent low levels of CIN. At the same time, we show that genomic alterations acquired in immortalised genome-unstable vHMECs are a mixture of random and fixed chromosomal rearrangements that could be potential sources of oncogenic changes and malignant evolution.

## 2. Results

### 2.1. Establishment of Immortalised and Non-Immortalised vHMECs with Different Cell Cycle Settings

**Figure 1:**
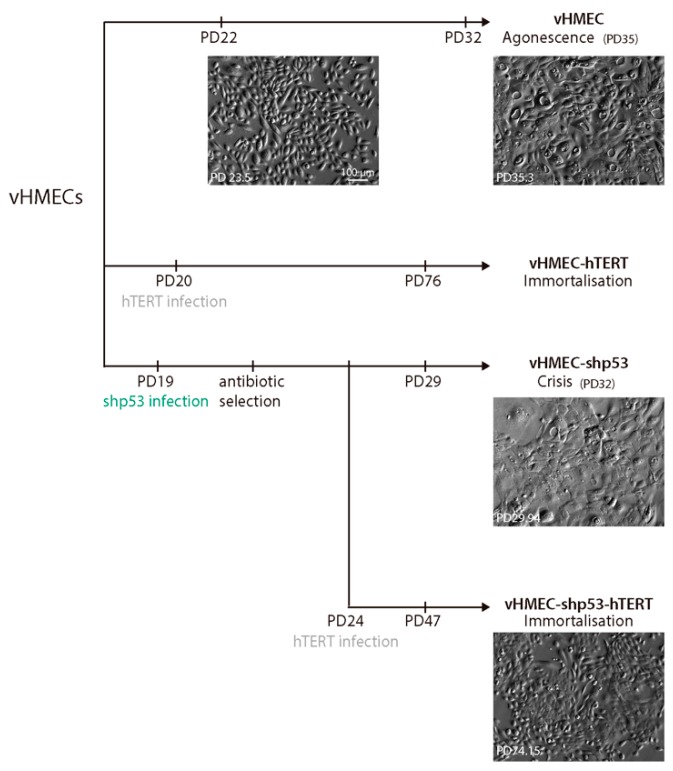
Scheme of the generation and analysis of different vHMEC cell lines. Young vHMECs were immortalised by transduction with hTERT containing lentivirus at PD20 to generate immortalised vHMECs. In addition, young vHMECs at PD19 were also infected with lentiviral particles containing the short hairpin RNA of p53 under the hU6 constitutive promoter to generate p53 compromised finite vHMECs. After a period of selection with puromycin, the cells were expanded and subsequently immortalised with the hTERT lentivirus at PD24. Cytogenetic analysis was performed at PD22 and PD32 for young and aged vHMECs, respectively. Immortalised vHMECs (vHMEC-hTERT) were karyotyped at PD76 and at PD130 (not shown). Finite but p53-deficient vHMECs (vHMEC-shp53) were analysed at PD29 and the immortalised cell line derivative (vHMEC-shp53-hTERT) at PD47. Phase contrast images of the different cell lines at different PD are shown. Scale bar corresponds to 100 µm.

To study how telomerase and p53 modulate the development and maintenance of CIN in human epithelial cells, p16^INK4a^-deficient HMECs (vHMECs) were genetically modified through lentivirus infection. Genetic modifications consisted of the generation of an immortalised vHMEC cell line by ectopical expression of the catalytic subunit of telomerase (Figure 1). hTERT immortalisation was performed at an early population doubling (PD) before vHMECs developed CIN associated with extensive telomere shortening. On the other hand, young vHMECs were also subjected to constitutive abrogation of p53 through short-hairpin p53 RNA lentiviral particles (Figure 1). After five PD, immortalisation of p53-deficient vHMECs was conducted through infection with the hTERT lentivirus (Figure 1).

**Figure 2:**
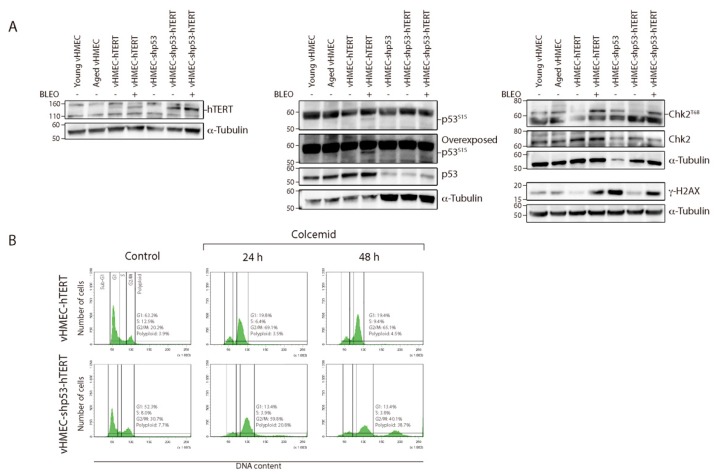
Validation of hTERT immortalisation and p53 downregulation in the different vHMECs. (**A**) Immunoblots of untreated cell lines as well as Bleocin™-treated p53-proficient and deficient immortalised vHMECs. Expression of the catalytic subunit of telomerase (hTERT) was observed at approximately 120 kDa only in the immortalised cell lines. Please note that at 160 kDa there is an unspecific band. A higher expression of hTERT, i.e., stronger band, was observed in the p53-deficient derivatives. α-Tubulin was used as loading control. The same protein extracts were immunoblotted for p53. Diminished levels of p53 were observed in the shp53-vHMEC variants after α-Tubulin normalisation. In addition, the functionality of p53 was validated by checking for the presence of p53^S15^ after DSBs induction by Bleocin™. Only p53-proficient immortalised vHMECs showed enhanced p53^S15^ levels after DNA damage. α-Tubulin was used as loading control. The presence of DNA damage was determined in the different cell lines by blotting γ-H2AX, a marker of DSBs. After α-Tubulin normalisation, the γ-H2AX levels were observed to increase in finite vHMECs concomitant to increasing telomere shortening and specifically when p53 function was abrogated. hTERT immortalisation reduced γ-H2AX levels in both p53-proficient and deficient vHMECs, but in vHMEC-shp53-hTERT DSBs still remained, as the level of γ-H2AX was comparable to that of telomere-compromised vHMECs. Similarly, Chk2^T68^, another marker of DSBs, was noticed in the finite and the Bleocin™-treated immortalised cells; (**B**) Representative cell cycle profiles of vHMEC-hTERT and vHMEC-shp53-hTERT cell lines 24 h and 48 h after colcemid treatment, as well as their respective controls. Cell cycle profiles remained stable throughout the time of the experiment for untreated cells. Colcemid treatment produced an accumulation of cells in the G2/M phase in vHMEC-hTERT. In the p53 compromised cells there was, in addition to the G2/M increase, an accumulation of cycling polyploids, i.e., more than 4N DNA content, specifically at 48 h after treatment. Cell cycle phases are marked and values indicated. A minimum of 10,000 cells were analysed per experiment.

Validation of the genetic modifications was performed by different approaches. Telomerase levels in the different cell lines were tested through hTERT expression by western blotting (Figure 2A), rather than using highly sensitive molecular methodologies such as RT-PCR of hTERT mRNA or RQ-TRAP. Only the cell lines transduced with hTERT showed a clear band for the catalytic subunit of telomerase, thus validating that cell immortalisation took place. In addition, inactivation of the p53 pathway through shRNA was confirmed by the reduced levels of p53 protein and by the fact that increased levels of p53^S15^ were unnoticed after cell exposure of vHMEC-shp53 cells to the DSBs inducer Bleocin™ (Figure 2A).

In addition, the functional status of p53 was determined by assaying the ability of cells to arrest growth after exposure to the microtubule destabilising agent colcemid. Microtubule inhibitors, such as colcemid or nocodazole, physically interfere with microtubule formation and activate the spindle assembly checkpoint (SAC) [34]. This checkpoint monitors kinetochore attachment [35] and delays chromatid separation and exit from mitosis until all kinetochores are saturated with and stably attached to spindle microtubules [36]. As for other checkpoints, an active SAC is not normally capable of blocking exit from mitosis indefinitely. Indeed, cells can evade the mitotic arrest and proceed to the next interphase without chromosome segregation by means of a process termed mitotic slippage or checkpoint adaptation. Under normal conditions, these cells with a tetraploid DNA content often suffer a long-lasting arrest at G1, most likely due to the induction of cellular senescence [37]. In contrast, polyploid cells that lack functional p53 have an increased ability to re-enter the cell cycle and initiate another round of DNA replication [38,39,40,41]. In order to determine if this was the case for our p53-deficient cells, FACS analysis of DNA content was performed after sustained exposure to 50 ng/mL of colcemid during 24 h or 48 h. Following 24 h exposure, there was an increase in cells in the G2/M fraction and a lower number of cells in G1, in both proficient and p53-deficient vHMEC-hTERT cells (Figure 2B), probably reflecting the accumulation of mitotically arrested cells. In contrast, FACS analysis of cells after sustained 48 h colcemid treatment demonstrated the presence of cells with an 8N DNA content compatible with cycling polyploids only in vHMEC-shp53-hTERT cells (Figure 2B).

In summary, we have efficiently generated p53-proficient and deficient mortal and immortal vHMEC lines from one individual to investigate the contribution of p53 and telomere status in the karyotypic evolution of epithelial human cells.

### 2.2. The Negative Impact of Telomere-Erosion on the Karyotype of vHMECs Is Enhanced by Targeted p53 Inactivation

Previous studies on vHMECs have shown that hypermethylation of the CDKN2A promoter allows the proliferation of breast cells carrying extremely short telomeres as well as uncapped chromosomes [29,30]. A direct link between exaggerated telomere shortening and chromosomal aberration formation has been obtained in several different human epithelial cell models [42,43,44,45]. To determine whether loss of p53 contributes to the intensification of the telomere-dependent CIN, we first evaluated the karyotype aberrations in unmodified vHMECs at an early culture stage (PD22) just after a period of selection when clones with p16^INK4a^ inactivation acquire proliferation capacity. In addition, a late culture stage (PD32) was analysed to detect abnormalities occurring over time before vHMECs cease proliferation by entering agonescence at approximately PD35 (Figure 1).

**Figure 3:**
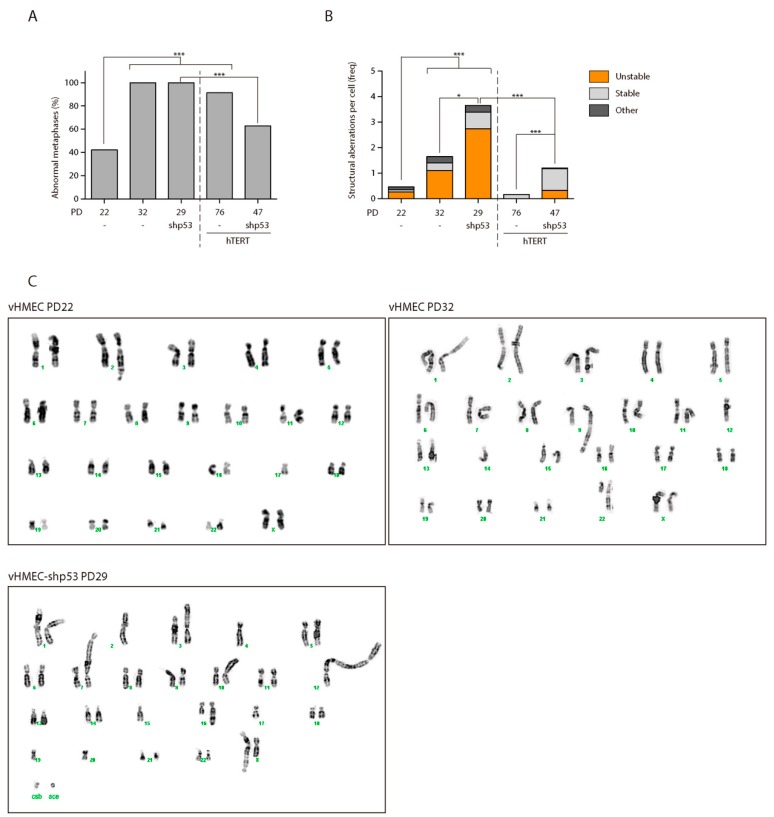
Cytogenetic analysis of the different cell lines. (**A**) Graph displaying the contribution of the telomere status and p53 functionality in the presence of abnormal karyotypes in vHMEC-derived cell lines. Statistical significance after Fisher’s exact test comparisons is shown. *** indicates *p*-values lower than 0.001; (**B**) Distribution of the different types of chromosomal structural aberrations: unstable, i.e., fusions or dicentric chromosomes and acentric fragments; stable, i.e., non-reciprocal and reciprocal translocations, isochromosomes, marker chromosomes and deletions; and other, i.e., chromosome and chromatid breaks. Statistical significance after Kruskal-Wallis test and Dunn’s multiple comparison post-test is shown. * indicates *p*-values lower than 0.05; *** indicates *p*-values lower than 0.001; (**C**) Example of finite vHMEC karyotypes. At PD22, young vHMECs show 45, XX, fus (2q;17q); at PD32, aged vHMECs show 45, XX, fus (9q;12q), nrt (22q;14q). By contrast, the karyotype of p53 compromised vHMECs demonstrates the complexity of their karyotype. At PD29, vHMEC-shp53 show 40, X, dic (2p;?;12p), nrt (3p;?), dic (4q;7p), dic (10p;?), nrt (16q;?), tetrac (17q;22;X;20p), +ace, +csb.

Cytogenetic analysis of vHMEC cells was performed using both inverted 4′,6-Diamidino-2-phenylindole dihydrochloride (DAPI) staining and pantelomeric-pancentromeric hybridisation with PNA probes. A total of 26 early passage vHMECs were karyotyped (Table S1). Eleven metaphase cells (42.31%) had an abnormal karyotype (Table 1 and Figure 3A,C). Structural chromosomal aberrations observed were chromosome fusions (fus) or dicentric chromosome (dic) (6 cells), non-reciprocal translocations (nrt) (2 cells), chromatid breaks (ctb) (3 cells) and acentric fragments (ace) (1 cell). The karyotype analysis of in vitro aged vHMECs metaphases after ten PDs (PD32) demonstrated the significant accumulation of aberrant cells with proliferation in the absence of telomerase (Table 1 and Figure 3A,C) (Fisher’s exact test, *p* < 0.0001). All aged cells were karyotypically abnormal (100%) (Table S2). The aberration most often observed was the presence of fus or dic (17 cells). Other aberrations were nrt (4 cells), isochromosome (i) (1 cell) and centric fragments (4 cells). Altogether, the accrual of telomere dysfunction in vHMECs results in highly structural rearranged karyotypes with increasing frequency of structural aberrations per cell (Table 2 and Figure 3B) (Kruskal-Wallis test, *p* < 0.0001). Of relevance, end-to-end chromosome fusions, a marker of dysfunctional telomeres, increased with PDs from 0.23 per cell in young vHMECs to 1.1 per cell in the aged vHMECs. None of the fusions observed in our cell lines presented interstitial telomeres at the junction point (Figure S1), and most of the fusion events were located at the chromosome terminus. These results point to telomere attrition, and not to the breakdown of the t-loop due to shelterin problems at the origin of end-to-end fusions.

**Figure 4:**
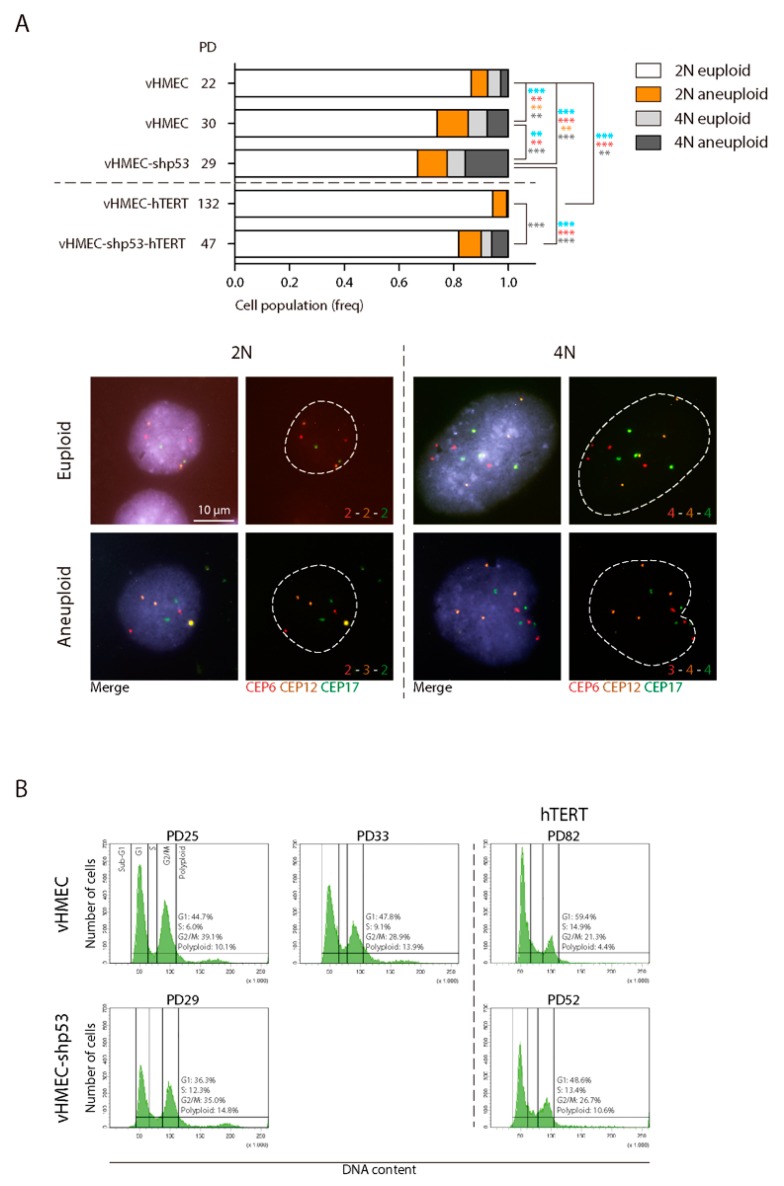
Analysis of chromosome number abnormalities. (**A**) Graph showing the frequency of euploid and aneuploid 2N and 4N among vHMECs after hybridisation with centromeric specific probes for chromosome 6 (CEP6), 12 (CEP12) and 17 (CEP17). Chi^2^ test demonstrated a significant increase in cells containing numerical aberrations (blue asterisks). Moreover, tetraploidisation events significantly increased in finite vHMECs with increasing telomere dysfunction and were aggravated when p53 was compromised (Fisher’s exact test, red asterisks). Statistical significance after Fisher’s exact test comparisons regarding 2N aneuploid and 4N aneuploid cells with asterisks in the same colour code as the legend is shown, and only *p*-values lower than 0.0125 were considered significant. ** indicates *p*-values lower than 0.01; *** indicates *p*-values lower than 0.001. Representative images of diploid and tetraploid cells with euploid and aneuploid configurations of tested centromeric probes are depicted. Scale bar corresponds to 10 µm; (**B**) Representative cell cycle profiles of vHMEC cell lines. FACs analysis also demonstrated the increase of polyploid cells concomitant with PDs in finite vHMECs. Immortalisation of young vHMECs did not engender tetraploids, whereas immortalisation of p53-compromised vHMECs resulted in an average 10% proportion of cycling polyploids. Cell cycle phases are marked and values indicated.

**Figure 5:**
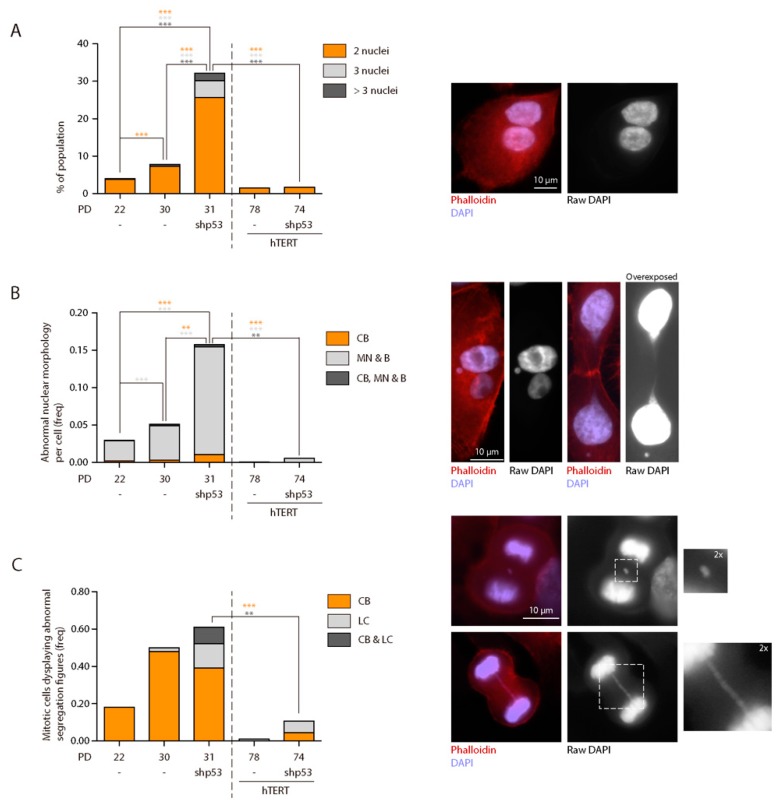
Analysis of abnormal morphologies in vHMEC derivatives. (**A**) Graph showing the percentage of binucleated, trinucleated cells and cells with more than three nuclei in interphase vHMECs. Finite vHMECs showed a significant increase in binucleation with increasing telomere attrition, and this effect was exacerbated when p53 was compromised. Representative images of a binucleated cell are shown. Scale bar corresponds to 10 µm; (**B**) Analysis of abnormal nuclear morphologies, i.e., chromatin bridges (CB), nuclear buds (B) and micronuclei (MN) among the different cell lines. Micronuclei and buds were the most frequent aberrations in vHMECs suffering telomere-dysfunction, followed by chromatin bridges. These aberrations were extremely abundant when p53 was compromised in finite vHMECs. Representative images of interphase cells displaying abnormal nuclear morphologies are shown. Scale bar corresponds to 10 µm; (**C**) Abnormal segregating figures were also observed in mitotic cells. In particular, chromatin bridges (CB) were abundant in those cells showing telomere-dysfunction. Likewise, those cells deficient for p53 were more prone to display lagging chromatin (LC). Representative images of abnormal anatelophases are shown. Scale bar corresponds to 10 µm. In the three graphs, statistical significance after Fisher’s exact test comparisons is shown with asterisks in the same colour code as the legend. Only *p*-values lower than 0.0125 after Bonferroni *p*-value correction, were considered significant. ** indicates *p*-values lower than 0.01; *** indicates *p*-values lower than 0.001

Besides structural chromosomal aberrations, numerical aberrations were evaluated through oligoFISH labelling of centromere-specific probes in interphase nuclei. This avoids artefactual chromosome loss due to the chromosome spreading technique. The FISH signals distribution of chromosome 6 (CEP6), 12 (CEP12) and 17 (CEP17) was scored in a minimum of 390 cells per condition (Table 3). At early PD, aneuploidy levels among diploid vHMECs were around 6% (Figure 4A) and, in agreement with published reports [46], increased significantly with PDs (Fisher’s exact test, *p* = 0.0057). Furthermore, given the already defined tetraploidisation effect of telomere dysfunction in vHMECs and other cell types [47,48], we also evaluated the extent of tetraploid cells in telomere-compromised vHMECs. The oligoFISH scoring of vHMECs demonstrated a significant accumulation of 4N cells with PDs (7.65% vs. 14.73% in vHMECs at PD22 and PD30, respectively; *p* = 0.0015, Fisher’s exact test) (Table 3 and Figure 4A). This increase in cell ploidy was also demonstrated by cytometric analysis where a minimum of 10,000 cells were evaluated per condition (10.1% vs. 13.9% in vHMECs at PD25 and PD33, respectively) (Figure 4B). Specifically, telomere dysfunction has been envisaged as a factor capable of interfering with the completion of cytokinesis through chromatin bridges emerging from end-to-end chromosome fusions [48]. For this purpose, mono- and multinucleated cells were also scored in vHMECs. After applying Texas Red-X Phalloidin to detect the cell cortex and DAPI staining to counterstain DNA, the analyses confirmed a significant increase in the frequency of binucleated cells with the accrual of telomere dysfunction (Fisher’s exact test, *p* < 0.0001) (Figure 5A).

Loss of p53 function may contribute to malignant progression by allowing the proliferation of cells with increased genomic instability. To determine if this was the case in our vHMEC line, a total of 23 vHMEC-shp53 cells were karyotyped at PD29, after shRNA infection (PD19), selection and subsequent cell expansion (Table S3). Similar to late passage p53-proficient vHMECs, no vHMEC-shp53 cell had a normal diploid karyotype (Table 1 and Figure 3A). However, the karyotype complexity was more pronounced in cells lacking p53 function, as the number of structural aberrations per cell increased extensively when p53 function was abrogated (3.65 vs. 1.65 aberrations/cell in vHMEC-shp53 PD29 and vHMEC PD32, respectively; Kruskal-Wallis test, *p* < 0.0001) (Table 2 and Figure 3B,C). Specifically, in p53-deficient vHMECs, there was an increase in marker chromosomes, as the highly reorganised karyotype made more difficult chromosome bands identification. The predominant types of structural changes were fused chromosomes in the form of dic or tricentric, followed by nrt and fragments, either centric or acentric. The analysis of the junction point of fusion events in multicentric chromosomes also demonstrated the absence of telomeric DNA by PNA hybridisation (Figure S1). Of relevance, the dicentric chromosomes in p53-deficient vHMECs were sometimes accompanied by acentric fragments, the consequence of creating chromosome breaks, thus denoting that telomere-shortening was not the only source for dicentric formation in this cell line. In addition, given the major role of p53 in the prevention of tetraploidy by activating apoptosis [49,50], its absence facilitated the generation and survival of tetraploid vHMECs. Although the rise in the polyploid population was not clearly envisaged through FACS analysis, probably by an accumulation of tetraploid cells in G1 (Figure 4B), the oligoFISH analysis demonstrated a significant increase in polyploid cells with the absence of p53 function when comparing both with young or aged vHMECs (Fisher’s exact test, *p* < 0.0001 and *p* = 0.0047, respectively) (Table 3 and Figure 4A). This data was also supported by the fourfold increase in multinucleated interphase cells in compromised p53 cells as compared to p53-proficient late passage vHMEC (Fisher’s exact test, *p* < 0.0001) (Figure 5A).

It should be noted that the Phalloidin-DAPI analysis allowed the identification of an elevated number of incorrectly aligned chromosomes at metaphase and lagging chromatin between segregating complements during anaphase, as well as micronuclei and buds in interphase cells lacking p53 function (Figure 5B,C). These improper chromosome distributions might result in unequal chromatid segregation among daughter cells, thus constituting a prominent source of aneuploidy. Indeed, centromeric-specific FISH scoring demonstrated an overall significant increase in chromosome number aberrations in shp53-deficient vHMECs (Chi^2^ test, *p* < 0.0001) (Figure 4A). In addition, these aneuploid configurations were extremely high among the 4N fraction of vHMEC-shp53 cells (Fisher’s exact test, *p* < 0.0001 and *p* = 0.0003, compared to young and aged vHMECs respectively) (Table 3 and Figure 4A). These observations, together with the minor fraction of multipolar divisions observed, suggest that extra centrosomes in p53-deficient tetraploid vHMECs induce transient multipolar spindles that could significantly increase the incidence of merotelic attachments and chromosome mis-segregation rates [51,52].

As a whole, progressive telomere dysfunction in vHMECs concomitantly increased the level of chromosomal aberrations, which further expanded in the absence of p53. Of relevance, whereas chromosome aberrations in telomere-compromised vHMECs predominantly affected specific chromosomes (Tables S1 and S2), nearly all the chromosomes participated in structural genome changes when p53 function was abolished (Table S3). Notably, all vHMECs either proficient or deficient for p53 ceased proliferation around PD30 without the emergence of spontaneous immortalised cells, thus indicating that abrogation of p53 function is insufficient to immortalise vHMECs, as has been previously described [33].

### 2.3. Absence of CIN When hTERT Is Ectopically Expressed in p53-Proficient Young vHMECs 

With the aim of generating pre-malignant immortalised cells, we ectopically expressed telomerase in telomere-compromised vHMECs. We assumed that if telomere-dependent BFB-cycles had begun, immortalised cells would maintain some ongoing instability. However, the immortalisation of aged vHMECs was not successful in this cell line as aged vHMECs did not overcome the agonescence limit (PD35) upon hTERT DNA virus delivery. Similarly, it was also not accomplished in late passages of vHMECs derived from other donors (unpublished results). In contrast, ectopic expression of hTERT was successful when infection took place at early passage vHMECs (PD20). Even in the absence of an antibiotic selection procedure, the cells resumed proliferation beyond the agonescence limit. This observation, together with the corroborated hTERT expression by western blotting (Figure 2A), confirmed that immortalisation had taken place.

**Figure 6:**
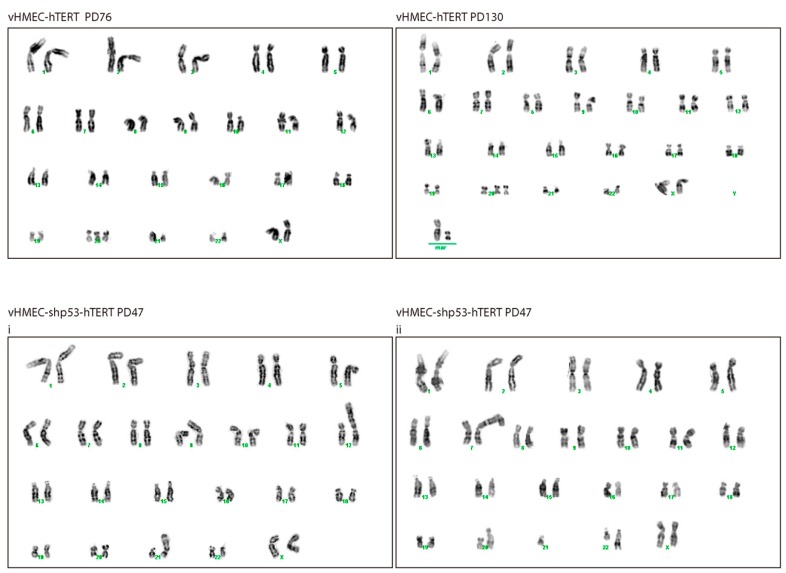
Karyotypes of immortalised p53-proficient and deficient vHMECs. Immortalisation of young vHMECs resulted in few chromosomal aberrations; the karyotype shown at PD76 is 47, XX, del (10p), +20. When the immortalised vHMECs were again karyotyped after 54 PDs (PD130), in addition to trisomy 20, two clonal marker chromosomes were observed. By contrast, immortalisation of p53-compromised vHMECs resulted in the accumulation of stable and unstable structural chromosome aberrations. Two karyotypes are shown: (**i**) 46, XX, nrt (9p;?), nrt (12p;?), dic (21p;?) and (**ii**) 45, XX, idic (7p;7p), +7, dic (20p;?), tric (22q;?;?), −21.

Examination of the vHMEC hTERT-immortalised cells by cytogenetic analyses at PD76 showed an elevated frequency of aberrant metaphases with an approximately diploid complement of chromosomes (Table 1 and Figure 3A and Figure 6) (Fisher’s exact test, *p*-value < 0.0001). In contrast to telomerase-deficient cells, vHMEC-hTERT cells showed predominantly clonal numerical chromosomal aberrations in the form of trisomy 20 alone (73.9%) or in combination with structural chromosomal aberrations (10.9%) (Table S4). Consistent with vHMEC immortalisation before telomeres became compromised, unstable aberrations such as fus or dic chromosomes were not observed (Figure 3B and Figure 6; Figure S2). The minor structural aberrations detected were nrt and deletions of unspecific chromosomes. In agreement with our observations, chromosome 20 trisomy has been reported to occur after ectopically hTERT expression in pre-stasis [53] and post-stasis HMECs [54,55], as well as in HMECs where p16^INK4a^ was abrogated through shRNA, BMI-1 [56] or CDK4^R24C^ mutation [57]. Moreover, these studies revealed the association of trisomy 20 with other chromosomal aberrations such as partial or complete trisomy of chromosomes 1q, 5, 7, 8q, 13, 16 and 18 [53,54,55,56,57]. Indeed, further karyotypic analysis of the vHMEC-hTERT at PD130, revealed in addition to trisomy 20, the gain of an extra marker chromosome 20 and an undefined marker chromosome (Figure 6) and/or two nrt, one involving 1q and the other involving 3q.

OligoFISH analysis did not reveal abnormal chromosome distribution as a major characteristic of early immortalised vHMECs (Table 3 and Figure 4A), or the presence of polyploid cells (Table 3 and Figure 4). These observances thus corroborate the defined role of telomere dysfunction in the generation of numerical chromosomal aberrations, as the stabilisation of the telomere length abolished the presence of chromatin bridges (Figure 5B,C), the intermediate structures that act for the generation of telomere-dependent CIN, as well as multinucleation events (Figure 5A).

As a whole, ectopic hTERT expression in young vHMECs efficiently immortalised the cells before telomere dysfunction triggered BFB-cycles. Telomerase immortalisation resulted in a relatively stable karyotype, mainly displaying aberrations involving chromosome 20. Moreover, during the 140 PDs the cells were continuously cultured in vitro, they evolved karyotypically, accumulating further chromosome aberrations that could be needed to improve the survival of the immortalised cells.

### 2.4. Reduced But Persistent CIN in p53-Deficient vHMECs Immortalised with hTERT

Given that hTERT immortalisation of vHMECs only resulted when lentivirus infection took place in young cells (PD20), we were concerned about the possibility of immortalising p53-deficient vHMECs. If the p53 loss and immortalisation events occur prior to attaining critically short telomeres, it is probable that p53 loss may not necessarily confer genomic instability. Indeed, abrogation of p53 in young vHMEC-hTERT from a different donor did not result in increased cytogenetic aberrations (unpublished results). Likewise, normal vHMECs stably transduced first with hTERT and afterwards with a shRNA against p53, proliferated indefinitely and did not show gross chromosomal alterations [58]. In addition, this also occurred in HCT116 colon tumour-derived immortal human cells [59].

Infection of vHMEC-shp53 with hTERT lentiviral particles at PD24, successfully resulted in immortalised p53-deficient vHMECs. Validation of hTERT expression was assessed by western blotting (Figure 2A) but the confirmation became clear through the active proliferation of vHMEC-shp53-hTERT beyond the crisis barrier and by the absence of a dying morphological appearance (Figure 1). Whereas finite p53-deficient vHMECs stopped proliferation at approximately PD32, the vHMEC-shp53-hTERTs maintained continuous proliferation with no morphological signs of growth defects until at least PD74 (Figure 1). On the whole, although p53 deficiency was not required for immortalisation, inactivation of p53 promoted immortalisation of more aged telomere-compromised vHMECs (PD20 vs. PD24).

The cytogenetic analysis of the vHMEC-shp53-hTERT at PD47 demonstrated that immortalisation significantly reduced the percentage of cells displaying chromosome aberrations when compared to p53-deficient vHMECs (Fisher’s exact test, *p* = 0.0004) (Table 1 and Figure 3A), but almost two thirds of cells still displayed an abnormal karyotype. When compared to vHMEC-hTERT, the p53-deficient immortalised cells presented a significantly-increased number of aberrations per cell (Kruskal-Wallis test, *p* < 0.0001) (Figure 3B). In addition, stabilisation of telomere ends in a p53-deficient settling statistically increased the frequency of stable chromosome aberrations when compared to non-immortalised cell lines (Fisher’s exact test, *p* = 0.0007; *p* < 0.0001 and *p* < 0.0001, when compared to young, aged and shp53-deficent vHMECs respectively) (Table 2). These aberrations were mainly non-reciprocal translocations, some of which were fixed on the karyotype and were shared by different cells (Table S5). In addition, although unstable aberrations significantly decreased with immortalisation (Fisher’s exact test, *p* = 0.0008) (Figure 3B), fused chromosomes (16.67% of metaphases) and centric or acentric chromosome fragments, signs of ongoing BFB-cycles, were still detected in immortalised p53-deficient vHMECs (Figure 6 and Figure S3). Accordingly, western blot analysis of phosphorylated H2AX at S139 (γ-H2AX), a hallmark of DSBs [60], confirmed a remaining fraction of DSBs in vHMEC-shp53-hTERT after α-Tubulin normalisation (Figure 2A). Whereas γ-H2AX levels increased in finite vHMECs concomitant to PDs and to p53-deficiencies, a reduction of DSBs was observed when p53-proficient vHMECs were immortalised with hTERT (Figure 2A). Of relevance, the level of γ-H2AX signalling in vHMEC-shp53-hTERT was comparable to that of telomere-compromised vHMECs, thus validating the cytogenetic results. The presence of DSBs was further evaluated by the presence of the activated form of the serine/threonine kinase Chk2, a key component of the DNA damage response. Chk2^T68^ was detected in all cell lines except the immortalised ones, thus demonstrating a reduction in DNA damage with cell immortalisation (Figure 2A).

Persistent chromosomal aberrations were not only of a structural type, as numerical aberrations were also scored in the vHMEC-shp53-hTERT cells. Immortalisation of p53 deficient vHMECs significantly reduced the overall frequency of numerical aberrations, as well as the frequency of polyploids (Chi^2^ and Fisher’s exact test, *p* < 0.0001) (Figure 4). Even so, the survival of unstable tetraploids was promoted as there was a significant fraction of polyploid cells with aneuploid configurations in comparison with vHMEC-hTERT (Table 3 and Figure 4A).

In sum, hTERT expression in p53-compromised vHMECs after BFB cycles are initiated results in the generation of an immortalised cell line that exhibits low CIN levels. This remaining CIN results in the heterogeneous presence of structural, numerical and ploidy aberrations and is visualised by the exhibition of anaphase bridges as well as lagging chromatin during cell division.

## 3. Discussion

In vivo studies in the mouse have revealed the constraining or promoting role of telomeres in cancer development. This dual capacity depends on the genetic context where dysfunctional telomeres occur, as highlighted by their enhanced tumorigenic potential when p53 is co-deleted [17,18,61]. Besides that, telomerase reactivation in the setting of a pre-existing telomere-induced genome instability period is required to actively drive carcinogenesis [19]. In humans, the finding of highly recurrent activating mutations in the hTERT gene promoter [1], together with widespread p53 mutations in cancer [62], provide support for the idea that circumvention of a telomere-p53 checkpoint is also essential for carcinogenesis in humans.

In the breast, short telomeres and widespread genomic instability can first be observed in premalignant lesions such as ductal carcinoma in situ (DCIS) [20,22], a stage where the p53 and Rb pathways are usually inactivated [63,64,65], suggesting that these lesions develop from cells expressing insufficient telomerase for telomere length maintenance. Cultured human mammary epithelial cells (HMECs) derived from cosmetic reductions are used to provide a better understanding of the molecular mechanisms and interactions involved in breast cancer development. Seminal studies by Stampfer laboratory defined a model for senescence barriers in cultured HMECs that evidenced the unique features of breast human cells. HMECs obtained from tissue explants proliferate for a few PDs before entering a growth plateau. Nevertheless, and in contrast to most epithelial cells, HMECs possess the ability to overcome this barrier owing to p16^INK4a^ promoter inactivation [29]. Subsequently, vHMECs resume proliferation for additional PDs until progressive telomere dysfunction concomitantly generates an increasing level of chromosomal aberrations that ultimately becomes lethal to the cell [30]. Specifically, studies in vHMECs derived from different donors have illustrated how the formation of dicentric chromosomes set in motion BFB-cycles, a mechanism capable of producing rapid and widespread changes in gene dosage as well as complex structural rearrangements [31,46,48,66,67]. In this study, and coincident with these observations, an enhanced complexity of the karyotype occurred in the finite and p53-competent vHMECs as PDs increased, presumably because of concomitant telomere attrition. Although the telomere length was not monitored and could be a limitation of the study, PNA telomeric FISH analysis in the finite vHMECs demonstrated a gradual increase of chromosomes with shorter telomeres, most likely compromised, throughout the cell culture. Moreover, the abrogation of p53 function in these cells resulted, as already reported [68], in a higher accumulation of aberrations per cell and, in a growth defect at earlier times than p53-proficient vHMECs. Moreover, the abrogation of p53 function in these cells resulted, as already reported, in a higher accumulation of aberrations per cell [68] and in a growth defect at earlier times than p53-proficient vHMECs. Collectively, the autocatalytic nature of BFB-cycles during rampant telomere attrition massively scrambles the genome yielding a wide range of lesions. Notably, the abrogation of p53 likely exacerbated short-telomere driven instability by increasing the fraction of structural and numerical chromosomal aberrations per cell, but specifically promoting the incidence of tetraploids. Strikingly, unstable tetraploid cells are believed to contribute to oncogenesis [69,70]. The enhanced tumorigenic capacity of tetraploid cells [69,70,71,72,73,74,75] could be expedited by their increased tolerance to chromosome mis-segregation events [76]. In this scenario, it is envisaged that telomere dysfunction may trigger genomic instability by fuelling rearranged karyotypes where structural, numerical and ploidy aberrations coexist. Eventually, the cumulative effect of centrosome-clustering on the preceding unstable polyploids could lead to further cellular genome remodelling that might contribute to epithelial carcinogenesis. Rare immortalisation events, probably arising due to the generation of additional errors during the genetic instability period, have occasionally been documented [77,78,79]. Nevertheless, the extensive reorganization and ongoing CIN, in the telomere-compromised vHMEC lines studied ultimately threatened cell viability as both p53-proficient and deficient vHMECs finally enter a growth plateau without the emergence of immortal outgrowths. This is in accordance with the described evidence that the extent of mutations cannot increase endlessly without adversely affecting cell fitness [80,81,82], and implies the existence of a threshold of genomic instability that the cell could tolerate [83].

It is speculated that a brief, or at least transient, episode of genomic instability offered by dysfunctional telomeres would avoid the persistent mutator phenotype that hampers cell proliferation, but might allow for the appearance of some rare advantageous mutations that would be selected and eventually favour neoplastic progression. Among them, telomerase reactivation in those highly reorganised cells would somehow reduce genome instability to a level compatible with the rescue of the cellular fitness, thus providing a route for transformation. With the aim of generating pre-malignant immortalised vHMECs, we ectopically expressed telomerase catalytic subunit in p53-proficient and deficient cells. Human telomerase is minimally composed of two components, the telomerase reverse transcriptase (hTERT) protein and the telomerase RNA template component (hTR). In addition, given that hTR is ubiquitously expressed, hTERT is considered the rate-limiting component that determines telomerase activity. The attempt to immortalise telomere-compromised aged vHMECs was unsuccessful, but hTERT expression in young vHMECs before BFB-cycles were set in motion resulted in immortalised cells with a stable karyotype that mainly displayed aberrations involving chromosome 20. These aberrations have been observed in a variety of immortalised epithelial cells such as oesophageal, nasopharyngeal, bronchial, ovarian surface, uroepithelial or Meibomian gland among others [84,85,86,87,88]. Since immortalisation, in those studies, was induced not only through hTERT expression but through HPVE6E7 or SV40 virus [84,85,86,87,88], the non-random occurrence of chromosome 20q gains strongly suggests that this genetic aberration contributes to the cellular immortalisation process. By contrast, hTERT expression in telomere-compromised and already reorganised p53-deficient vHMECs was sufficient to sustain cell viability beyond the agonescent/crisis limit, but at the cost of reducing their intrinsic chromosome instability. Nevertheless, more than half of the hTERT immortalised vHMEC-shp53 cells still showed an abnormal karyotype, displaying both structural, numerical and ploidy aberrations. Notably, even though hTERT overexpression promoted a shift of chromosome aberrations towards stable-type, stabilisation of telomere ends did not, in any case, completely abolish the presence of unstable aberrations. Specifically, dicentric and tricentric chromosomes, as well as acentric fragments and deleted chromosomes, were evidenced in the metaphase plates of vHMEC-shp53-hTERT cells. Furthermore, the presence of lagging chromatin as well as chromatin bridges during cell division denoted actively ongoing BFB cycles and the more likely evolution of the karyotype.

As a whole, our results demonstrate that hTERT overexpression provides a route out of telomere crisis, as stabilisation of telomere ends rescued cellular fitness. However, only the immortalisation of cells that have progressed through a period of telomere-dependent CIN resulted in evolving karyotypes containing both fixed and random chromosomal aberrations. This persistent but reduced CIN would allow for further genome complexity and would thus facilitate the acquisition of the mutations needed to ultimately transform cells to malignity. Consistent with this, the generation of potentially neoplastic cells will occur when telomerase reactivation takes place after CIN has been triggered and before it reaches an intolerable level that leads to cell extinction. If this happens, telomere-dependent induced CIN can play a significant role in the generation of the karyotypic aberrations and the genomic instability observed in human breast carcinomas.

## 4. Materials and Methods

### 4.1. Cell Lines

Post-stasis variant human mammary epithelial cells (vHMECs) were obtained from Cell Applications Inc. (San Diego, CA, USA). vHMECs were cultured with serum-free MEpiCM medium supplemented with MEpiCGS and penicillin/streptomycin (all from ScienCell Research Laboratories, Carlsbad, CA, USA), or with M87AX [89]. Growth conditions were 5% CO_2_ and 37 °C. Culture population doublings (PDs) were calculated using the formula: PD = PD_initial_ + log_2_ (N_final_/N_initial_), where N_initial_ is the number of viable cells plated, and N_final_ is the number of viable cells harvested.

### 4.2. Lentiviral Vectors, Lentivirus Production and Transduction

The lentiviral construct for p53 short hairpin RNA (shp53 pLKO.1 puro) was from Dr Bob Weinberg (Addgene plasmid #19119) and the hTERT lentivirus was supplied by the Viral Vector Facility, CNIC, Madrid, Spain. To generate lentiviral particles, the psPAX2 and pMD2.G plasmids together with the plasmid containing the gene of interest, were introduced in HEK 293T packaging cells using Calphos Mammalian Transfection kit (Clontech, Mountain View, CA, USA). Supernatants were collected at 48 and 72 h post-transfection and concentrated using Amicon 100,000 centrifugal filter units (Merck-Millipore, Burlington, MA, USA).

### 4.3. Western Blotting

Proteins were extracted with 2% SDS, 67 mM Tris HCl (pH 6.8) containing protease and phosphatase inhibitors. Protein extracts were sonicated twice at 25% amplitude for 15 s, boiled at 95 °C for 15 min and centrifuged at 20,000 *g* for 10 min. Proteins were quantified using the BCA method and absorbance was read at 540 nm with a Victor3 spectrophotometer (PerkinElmer, Waltham, MA, USA). The proteins (30 µg) were separated using 10% acrylamide or 10% Bis-Tris gels (Life Technologies, Carlsbad, CA, USA, ThermoFisher Scientific, Waltham, MA, USA) at 35 mAmp and transferred onto nitrocellulose membranes at 30 V. Membranes were blocked with 5% non-fat milk or BSA. Primary antibodies used were: rabbit anti-hTERT (Rockland, 600-401-252S), rabbit anti-p53^S15^ (ThermoFisher Scientific, 14H61L24), mouse anti-p53 (ThermoFisher Scientific, DO-1), rabbit anti-Chk2^T68^ (Cell Signalling, 2661, Danvers, MA, USA), mouse anti-Chk2 (Millipore, 05-649) and mouse anti-γH2AX (Upstate; 07-164). The specificity of the hTERT antibody was validated in primary human mammary epithelial cells derived from reduction mammoplasty tissue at passage 2 (data not shown). Furthermore, mouse anti-α-Tubulin (Sigma, B-5-1-2, St. Louis, MO, USA) was used as loading control. Primary antibodies were incubated overnight at 4 °C. Secondary anti-mouse or anti-rabbit horseradish peroxidase (HRP) conjugated antibodies were used and incubated for 1 h at room temperature. Chemiluminescent detection was performed using HRP solution and luminol (Millipore), and images were acquired using Chemidoc, processed with Quantity One software and analysed with ImageLab™ 6.0.0 (BioRad, Hercules, CA, USA).

### 4.4. Drug Treatments

DSBs were generated in vHMECs and derivatives through exposure to the radiomimetic drug Bleocin™ (Calbiochem, Merck-Chemicals, Darmstadt, Germany), a bleomycin compound, at a final concentration of 2.5 µg/mL. The drug was washed out after 1 h exposure and the cells were left to recover for 60 min before protein extraction.

Colcemid (GIBCO) at a final concentration of 50 ng/mL was added to asynchronously proliferating p53-proficient and deficient vHMEC-hTERT cells. After 24 h of colcemid exposure, the cells were collected and fixed in 70% ethanol and kept frozen until FACS processing. Additional experiments consisted of 48 h colcemid treatment before fixation.

### 4.5. Obtaining Metaphase Cells and End-to-End Fusion Scoring Criteria

Exponentially-growing vHMEC cell lines were exposed to colcemid (0.5 µg/mL) for 2 h. Cells were trypsinised, swollen in 0.075 M KCl and fixed in methanol:acetic acid (3:1). Cell suspensions were dropped onto clean slides and stored at −20 °C until use. For end-fusion scoring purposes, slides were first stained with DAPI. Then the metaphase plates were captured, and the karyotype was performed by reverse DAPI staining, which results in a reproducible G band-like pattern that allows for accurate individual chromosome identification before the chromosomes become swollen by the denaturation step. Afterwards, the slides were hybridised with the PNA probes and the metaphases were relocated to analyse the telomere and centromere status of each chromosome. A fusion event was considered when the connection between chromatids (1 or 2) was verified on the initial DAPI stained image. This procedure reduces the possibility of end-fusion events being confused with mere alignment of chromosomes.

### 4.6. In Situ Fluorescence Hybridisation

Telomere and centromere PNA-FISH: Metaphase spreads were hybridised with pantelomeric (Rho-(CCCTAA)_3_, PE Biosystems, Foster City, CA, USA) and pancentromeric (FITC-AAACACTCTTTTTGTAGA, Panagene, Daejeon, South Korea) PNA probes. Denaturation took place at 80 °C for 3 min and hybridisation was performed at 37 °C for 2 h in a humid chamber. Afterwards, slides were washed twice with 70% formamide for 15 min, followed by three TNT (Trizma Base 50 mM, NaCl 150 mM and Tween-20 0.25%) washes for 5 min. Dehydrated slides were counterstained with DAPI.

OligoFISH: Interphase nuclei spreads were treated with pepsin-HCl at 37 °C for 10 min, post-fixed with formaldehyde-MgCl_2_ and denatured with 70% formamide at 74 °C. Specific centromeric probes for chromosomes 6 (Gold DY539), 12 (Red DY590) and 17 (Green DY490) (Cellay, Inc., Cambridge, MA, USA) were hybridised for 2 h in a humid chamber followed by one 5 min wash with 0.2 × SSC − 0.1% SDS at 50 °C and a 2 × SSC wash. Cells were dehydrated and counterstained with DAPI.

### 4.7. DAPI and Texas Red-X Phalloidin Staining

For the analysis of abnormal nuclear morphologies in interphase or mitosis, cells were cultured on coverslips and fixed with 4% paraformaldehyde for 10 min at 37 °C and rinsed twice in phosphate buffer solution (PBS). Then, cells were permeabilised with 1% Triton X-100 at room temperature during 10 min, rinsed briefly and stained for 5 min in 1 µL solution of Texas Red-X-Phalloidin [200 IU/mL] (Molecular probes) in 1 mL 1 × PBS − 0.1% Tween20-0.5% foetal calf serum. After two or more washes with PBS, coverslips were allowed to dry and counterstained with DAPI.

### 4.8. Fluorescent Microscopy and Fluorescent Images

Fluorescent staining was visualised under an Olympus BX60 microscope equipped with epifluorescent optics and a camera (Applied Imaging, Inc., Grand Rapids, MI, USA). The fluorochromes were visualised through simple filters and images were captured and analysed using Cytovision software (Applied Imaging, Inc.).

### 4.9. Flow Cytometry

For cell cycle analysis, the cells were harvested and fixed in 70% ethanol and kept at −20 °C until processing.

The fixed cells were permeabilised with 1 × PBS − 1%Triton X-100 solution and stained with propidium iodide solution (PBS − 1% Triton X-100, propidium iodide 45 µg/mL, and RNase 0.2 mg/mL) before cytometric processing. Analysis was performed under a FACSCalibur (Beckton Dickinson, Franklin Lakes, NJ, USA). Sample excitation was done with a 488 nm laser and a minimum of 10,000 events were collected per sample. Single cells were gated first by forward scatter (FSC) and side scatter (SSC), and DNA content of single cells was measured on FL3 (670 nm long pass filter) and plotted vs. number of cells. The data were analysed with BD FACSDiva software v7.0.

### 4.10. Statistical Analysis

Data analysis was carried out with GraphPad Prism version 5 software (GraphPad Software Inc., La Jolla, CA, USA). Normality distribution was tested by Saphiro-Wilk normality test. Data sets were compared using Chi^2^ test, Fisher’s exact test, and Kruskal-Wallis test followed by Dunn’s multiple comparison post-test. *p*-values less than 0.05 were considered significant. When multiple comparisons were made, the Bonferroni p-value correction was applied and only *p*-values lower than 0.0125 were considered significant.

## Figures and Tables

**Table 1:** Cytogenetic analysis in the different vHMEC cell lines.

Cell Line	PD	Analysed Cells n	Abnormal Cells % (n)	Cells with fus/dic % (n)	Cells with nrt % (n)	Cells with Structural AA % (n)	Cells with Clonal Numerical AA % (n)
vHMEC	22	26	42.31 (11)	23.08 (6)	7.69 (2)	42.31 (11)	0.00 (0)
vHMEC	32	20	100.00 (20)	85.00 (17)	20.00 (4)	100.00 (20)	0.00 (0)
vHMEC-shp53	29	23	100.00 (23)	82.61 (19)	21.74 (5)	100.00 (23)	0.00 (0)
vHMEC-hTERT	76	46	91.30 (42)	0.00 (0)	6.52 (3)	17.39 (8)	84.78 (39)
vHMEC-shp53-hTERT	47	54	62.96 (34)	16.67 (9)	50.00 (27)	62.96 (34)	0.00 (0)

PD: population doubling; n: number; AA: aberrations.

**Table 2:** Distribution of the types of structural chromosome aberrations.

Cell Line	PD	Analysed Cells n	Total AA/Cell Freq. (n)	UNSTABLE AA			STABLE AA		OTHER AA	
				fus/dic n	Ace n	AA/Cell Freq.	nrt/i/mar /del n	AA/Cell Freq.	csb/ctb n	AA/Cell Freq.
vHMEC	22	26	0.46 (12)	6	1	0.27	2	0.08	3	0.12
vHMEC	32	20	1.65 (33)	22	0	1.10	6	0.30	5	0.25
vHMEC-shp53	29	23	3.65 (84)	54	9	2.74	15	0.65	6	0.26
vHMEC-hTERT	76	46	0.17 (8)	0	0	0.00	8	0.17	0	0.00
vHMEC-shp53-hTERT	47	54	1.20 (65)	15	3	0.33	46	0.85	1	0.02

PD: population doubling; n: number; AA: aberrations.

**Table 3:** OligoFISH analysis of centromeric specific probes for chromosome 6, 12 and 17.

Cell Line	PD	Cells Analysed n	2N		4N		4N Fraction %
			Cells n	Aneuploidy Freq. (n)	Cells n	Aneuploidy Freq. (n)	
vHMEC	22	392	362	0.06 (23)	30	0.37 (11)	7.65
vHMEC	30	414	353	0.13 (47)	61	0.52 (32)	14.73
vHMEC-shp53	29	430	334	0.14 (47)	96	0.71 (68)	22.33
vHMEC-hTERT	132	846	841	0.05 (44)	5	0.00 (0)	0.59
vHMEC-shp53-hTERT	47	391	352	0.09 (32)	39	0.62 (24)	9.97

PD: population doubling; n: number.

## Supplementary Materials

Supplementary materials can be found at http://www.mdpi.com/1422-0067/19/7/2078/s1.

## References

[1] Barthel F.P., Wei W., Tang M., Martinez-Ledesma E., Hu X., Amin S.B., Akdemir K.C., Seth S., Song X., Wang Q. (2017). Systematic analysis of telomere length and somatic alterations in 31 cancer types. Nat. Genet..

[2] Hanahan D., Weinberg R.A. (2011). Hallmarks of cancer: The next generation. Cell.

[3] Michor F., Iwasa Y., Vogelstein B., Lengauer C., Nowak M.A. (2005). Can chromosomal instability initiate tumorigenesis?. Semin. Cancer Biol..

[4] Chen J., Fu L., Zhang L.Y., Kwong D.L., Yan L., Guan X.Y. (2012). Tumor suppressor genes on frequently deleted chromosome 3p in nasopharyngeal carcinoma. Chin. J. Cancer.

[5] Kops G.J., Weaver B.A., Cleveland D.W. (2005). On the road to cancer: Aneuploidy and the mitotic checkpoint. Nat. Rev. Cancer.

[6] Loeb L.A. (2011). Human cancers express mutator phenotypes: Origin, consequences and targeting. Nat. Rev. Cancer.

[7] Negrini S., Gorgoulis V.G., Halazonetis T.D. (2010). Genomic instability—An evolving hallmark of cancer. Nat. Rev. Mol. Cell Biol..

[8] Kolodner R.D., Cleveland D.W., Putnam C.D. (2011). Aneuploidy drives a mutator phenotype in cancer. Science.

[9] Thompson S.L., Bakhoum S.F., Compton D.A. (2010). Mechanisms of chromosomal instability. Curr. Biol..

[10] Olovnikov A.M. (1973). A theory of marginotomy: The incomplete copying of template margin in enzymic synthesis of polynucleotides and biological significance of the phenomenon. J. Theor. Biol..

[11] Daniali L., Benetos A., Susser E., Kark J.D., Labat C., Kimura M., Desai K., Granick M., Aviv A. (2013). Telomeres shorten at equivalent rates in somatic tissues of adults. Nat. Commun..

[12] Harley C.B., Futcher A.B., Greider C.W. (1990). Telomeres shorten during ageing of human fibroblasts. Nature.

[13] Takai H., Smogorzewska A., De Lange T. (2003). DNA damage foci at dysfunctional telomeres. Curr. Biol..

[14] Kaul Z., Cesare A.J., Huschtscha L.I., Neumann A.A., Reddel R.R. (2011). Five dysfunctional telomeres predict onset of senescence in human cells. EMBO Rep..

[15] D’Adda di Fagagna F., Reaper P.M., Clay-Farrace L., Fiegler H., Carr P., von Zglinicki T., Saretzki G., Carter N.P., Jackson S.P. (2003). A DNA damage checkpoint response in telomere-initiated senescence. Nature.

[16] Cesare A.J., Hayashi M.T., Crabbe L., Karlseder J. (2013). The telomere deprotection response is functionally distinct from the genomic DNA damage response. Mol. Cell.

[17] Chin L., Artandi S.E., Shen Q., Tam A., Lee S.L., Gottlieb G.J., Greider C.W., Depinho R.A. (1999). p53 deficiency rescues the adverse effects of telomere loss and cooperates with telomere dysfunction to accelerate carcinogenesis. Cell.

[18] Artandi S.E., Chang S., Lee S.L., Alson S., Gottlieb G.J., Chin L., DePinho R.A. (2000). Telomere dysfunction promotes non-reciprocal translocations and epithelial cancers in mice. Nature.

[19] Ding Z., Wu C.J., Jaskelioff M., Ivanova E., Kost-Alimova M., Protopopov A., Chu G.C., Wang G., Lu X., Labrot E.S. (2012). Telomerase reactivation following telomere dysfunction yields murine prostate tumors with bone metastases. Cell.

[20] Chin K., de Solorzano C.O., Knowles D., Jones A., Chou W., Rodriguez E.G., Kuo W.-L.L., Ljung B.-M.M., Chew K., Myambo K. (2004). In situ analyses of genome instability in breast cancer. Nat. Genet..

[21] Meeker A.K., Hicks J.L., Gabrielson E., Strauss W.M., De Marzo A.M., Argani P. (2004). Telomere shortening occurs in subsets of normal breast epithelium as well as in situ and invasive carcinoma. Am. J. Pathol..

[22] Meeker A.K., Argani P. (2004). Telomere shortening occurs early during breast tumorigenesis: A cause of chromosome destabilization underlying malignant transformation?. J. Mammary Gland Biol. Neoplasia.

[23] Tanaka H., Abe S., Huda N., Tu L., Beam M.J., Grimes B., Gilley D. (2012). Telomere fusions in early human breast carcinoma. Proc. Natl. Acad. Sci. USA.

[24] Ellsworth R.E., Blackburn H.L., Shriver C.D., Soon-Shiong P., Ellsworth D.L. (2017). Molecular heterogeneity in breast cancer: State of the science and implications for patient care. Semin. Cell Dev. Biol..

[25] Sugino T., Yoshida K., Bolodeoku J., Tahara H., Buley I., Manek S., Wells C., Goodison S., Ide T., Suzuki T. (1996). Telomerase activity in human breast cancer and benign breast lesions: Diagnostic applications in clinical specimens, including fine needle aspirates. Int. J. Cancer.

[26] Bednarek A.K., Sahin A., Brenner A.J., Johnston D.A., Aldaz C.M. (1997). Analysis of telomerase activity levels in breast cancer: Positive detection at the in situ breast carcinoma stage. Clin. Cancer Res..

[27] Poremba C., Shroyer K.R., Frost M., Diallo R., Fogt F., Schäfer K.L., Bürger H., Shroyer A.L., Dockhorn-Dworniczak B., Boecker W. (1999). Telomerase is a highly sensitive and specific molecular marker in fine-needle aspirates of breast lesions. J. Clin. Oncol..

[28] Shpitz B., Zimlichman S., Zemer R., Bomstein Y., Zehavi T., Liverant S., Bernehim J., Kaufman Z., Klein E., Shapira Y. (1999). Telomerase activity in ductal carcinoma in situ of the breast. Breast Cancer Res. Treat..

[29] Brenner A.J., Stampfer M.R., Aldaz C.M. (1998). Increased p16 expression with first senescence arrest in human mammary epithelial cells and extended growth capacity with p16 inactivation. Oncogene.

[30] Romanov S.R., Kozakiewicz B.K., Holst C.R., Stampfer M.R., Haupt L.M., Tlsty T.D. (2001). Normal human mammary epithelial cells spontaneously escape senescence and acquire genomic changes. Nature.

[31] Soler D., Genescà A., Arnedo G., Egozcue J., Tusell L. (2005). Telomere dysfunction drives chromosomal instability in human mammary epithelial cells. Genes Chromosom. Cancer.

[32] Genescà A., Pampalona J., Frías C., Domínguez D., Tusell L. (2011). Role of telomere dysfunction in genetic intratumor diversity. Adv. Cancer Res..

[33] Garbe J.C., Holst C.R., Bassett E., Tlsty T.D., Stampfer M.R. (2007). Inactivation of p53 function in cultured human mammary epithelial cells turns the telomere-length dependent senescence barrier from agonescence into crisis. Cell Cycle.

[34] Musacchio A., Salmon E.D. (2007). The spindle-assembly checkpoint in space and time. Nat. Rev. Mol. Cell Biol..

[35] Rieder C.L., Schultz A., Cole R., Sluder G. (1994). Anaphase onset in vertebrate somatic cells is controlled by a checkpoint that monitors sister kinetochore attachment to the spindle. J. Cell Biol..

[36] Rieder C.L., Cole R.W., Khodjakov A., Sluder G. (1995). The checkpoint delaying anaphase in response to chromosome monoorientation is mediated by an inhibitory signal produced by unattached kinetochores. J. Cell Biol..

[37] Andreassen P.R., Lohez O.D., Lacroix F.B., Margolis R.L. (2001). Tetraploid state induces p53-dependent arrest of nontransformed mammalian cells in G1. Mol. Biol. Cell.

[38] Borel F., Lohez O.D., Lacroix F.B., Margolis R.L. (2002). Multiple centrosomes arise from tetraploidy checkpoint failure and mitotic centrosome clusters in p53 and RB pocket protein-compromised cells. Proc. Natl. Acad. Sci. USA.

[39] Casenghi M., Mangiacasale R., Tuynder M., Caillet-Fauquet P., Elhajouji A., Lavia P., Mousset S., Kirsch-Volders M., Cundari E. (1999). p53-independent apoptosis and p53-dependent block of DNA rereplication following mitotic spindle inhibition in human cells. Exp. Cell Res..

[40] Cross S.M., Sanchez C.A., Morgan C.A., Schimke M.K., Ramel S., Idzerda R.L., Raskind W.H., Reid B.J. (1995). A p53-dependent mouse spindle checkpoint. Science.

[41] Vogel C., Kienitz A., Hofmann I., Müller R., Bastians H. (2004). Crosstalk of the mitotic spindle assembly checkpoint with p53 to prevent polyploidy. Oncogene.

[42] Deng W., Tsao S.W., Guan X.Y., Lucas J.N., Cheung A.L.M. (2003). Role of short telomeres in inducing preferential chromosomal aberrations in human ovarian surface epithelial cells: A combined telomere quantitative fluorescence in situ hybridization and whole-chromosome painting study. Genes Chromosom. Cancer.

[43] Deng W., Tsao S.W., Guan X.Y., Lucas J.N., Si H.X., Leung C.S., Mak P., Wang L.D., Cheung A.L.M. (2004). Distinct profiles of critically short telomeres are a key determinant of different chromosome aberrations in immortalized human cells: Whole-genome evidence from multiple cell lines. Oncogene.

[44] Der-Sarkissian H., Bacchetti S., Cazes L., Londoño-Vallejo J.A. (2004). The shortest telomeres drive karyotype evolution in transformed cells. Oncogene.

[45] Plug-DeMaggio A.W., Sundsvold T., Wurscher M.A., Koop J.I., Klingelhutz A.J., McDougall J.K. (2004). Telomere erosion and chromosomal instability in cells expressing the HPV oncogene 16E6. Oncogene.

[46] Pampalona J., Soler D., Genescà A., Tusell L. (2010). Whole chromosome loss is promoted by telomere dysfunction in primary cells. Genes Chromosom. Cancer.

[47] Davoli T., Denchi E.L., de Lange T. (2010). Persistent telomere damage induces bypass of mitosis and tetraploidy. Cell.

[48] Pampalona J., Frías C., Genescà A., Tusell L. (2012). Progressive telomere dysfunction causes cytokinesis failure and leads to the accumulation of polyploid cells. PLoS Genet..

[49] Castedo M., Coquelle A., Vivet S., Vitale I., Kauffmann A., Dessen P., Pequignot M.O., Casares N., Valent A., Mouhamad S. (2006). Apoptosis regulation in tetraploid cancer cells. EMBO J..

[50] Senovilla L., Vitale I., Galluzzi L., Vivet S., Joza N., Younes A.B., Rello-Varona S., Castedo M., Kroemer G. (2009). p53 represses the polyploidization of primary mammary epithelial cells by activating apoptosis. Cell Cycle.

[51] Ganem N.J., Godinho S.A., Pellman D. (2009). A mechanism linking extra centrosomes to chromosomal instability. Nature.

[52] Silkworth W.T., Nardi I.K., Scholl L.M., Cimini D. (2009). Multipolar Spindle Pole Coalescence is a Major Source of Kinetochore Mis-Attachment and Chromosome Mis-Segregation in Cancer Cells. PLoS ONE.

[53] Garbe J.C., Vrba L., Sputova K., Fuchs L., Novak P., Brothman A.R., Jackson M., Chin K., LaBarge M.A., Watts G. (2014). Immortalization of normal human mammary epithelial cells in two steps by direct targeting of senescence barriers does not require gross genomic alterations. Cell Cycle.

[54] Toouli C., Huschtscha L., Neumann A. (2002). Comparison of human mammary epithelial cells immortalized by simian virus 40 T-Antigen or by the telomerase catalytic subunit. Oncogene.

[55] Rao K., Bryant E., O’Hara Larivee S., McDougall J.K. (2003). Production of spindle cell carcinoma by transduction of H-Ras 61L into immortalized human mammary epithelial cells. Cancer Lett..

[56] Haga K., Ohno S., Yugawa T., Narisawa-Saito M., Fujita M., Sakamoto M., Galloway D.A., Kiyono T. (2007). Efficient immortalization of primary human cells by p16^INK4a^-specific short hairpin RNA or Bmi-1, combined with introduction of hTERT. Cancer Sci..

[57] Joshi P.S., Modur V., Cheng J., Robinson K., Rao K. (2017). Characterization of immortalized human mammary epithelial cell line HMEC 2.6. Tumour Biol..

[58] Ulbricht U., Sommer A., Beckmann G., Lutzenberger M., Seidel H., Kreft B., Toschi L. (2013). Isogenic human mammary epithelial cell lines: Novel tools for target identification and validation. Comprehensive characterization of an isogenic human mammary epithelial cell model provides evidence for epithelial-mesenchymal transition. Breast Cancer Res. Treat..

[59] Bunz F., Fauth C., Speicher M.R., Dutriaux A., Sedivy J.M., Kinzler K.W., Vogelstein B., Lengauer C. (2002). Targeted inactivation of p53 in human cells does not result in aneuploidy. Cancer Res..

[60] Rogakou E.P., Pilch D.R., Orr A.H., Ivanova V.S., Bonner W.M. (1998). DNA double-stranded breaks induce histone H2AX phosphorylation on serine 139. J. Biol. Chem..

[61] Roake C.M., Artandi S.E. (2017). Control of Cellular Aging, Tissue Function, and Cancer by p53 Downstream of Telomeres. Cold Spring Harb. Perspect. Med..

[62] Olivier M., Hollstein M., Hainaut P. (2010). TP53 Mutations in Human Cancers: Origins, Consequences, and Clinical Use. Cold Spring Harb. Perspect. Biol..

[63] Holst C.R., Nuovo G.J., Esteller M., Chew K., Baylin S.B., Herman J.G., Tlsty T.D. (2003). Methylation of p16^INK4a^ promoters occurs in vivo in histologically normal human mammary epithelia. Cancer Res..

[64] Shackney S.E., Silverman J.F. (2003). Molecular evolutionary patterns in breast cancer. Adv. Anat. Pathol..

[65] Hui R., Macmillan R.D., Kenny F.S., Musgrove E.A., Blamey R.W., Nicholson R.I., Robertson J.F., Sutherland R.L. (2000). *INK4a* gene expression and methylation in primary breast cancer: Overexpression of p16^INK4a^ messenger RNA is a marker of poor prognosis. Clin. Cancer Res..

[66] Tusell L., Soler D., Agostini M., Pampalona J., Genescà A. (2008). The number of dysfunctional telomeres in a cell: One amplifies; more than one translocate. Cytogenet. Genome Res..

[67] Pampalona J., Roscioli E., Silkworth W.T., Bowden B., Genescà A., Tusell L., Cimini D. (2016). Chromosome Bridges Maintain Kinetochore-Microtubule Attachment throughout Mitosis and Rarely Break during Anaphase. PLoS ONE.

[68] Seewaldt V.L., Mrózek K., Sigle R., Dietze E.C., Heine K., Hockenbery D.M., Hobbs K.B., Caldwell L.E. (2001). Suppression of p53 function in normal human mammary epithelial cells increases sensitivity to extracellular matrix-induced apoptosis. J. Cell Biol..

[69] Fujiwara T., Bandi M., Nitta M., Ivanova E.V., Bronson R.T., Pellman D. (2005). Cytokinesis failure generating tetraploids promotes tumorigenesis in p53-null cells. Nature.

[70] Davoli T., de Lange T. (2012). Telomere-driven tetraploidization occurs in human cells undergoing crisis and promotes transformation of mouse cells. Cancer Cell.

[71] Duelli D.M., Padilla-Nash H.M., Berman D., Murphy K.M., Ried T., Lazebnik Y. (2007). A virus causes cancer by inducing massive chromosomal instability through cell fusion. Curr. Biol..

[72] Nguyen H.G., Makitalo M., Yang D., Chinnappan D., Hilaire C.S., Ravid K., St Hilaire C., Ravid K. (2009). Deregulated Aurora-B induced tetraploidy promotes tumorigenesis. FASEB J..

[73] Olaharski A.J., Sotelo R., Solorza-Luna G., Gonsebatt M.E., Guzman P., Mohar A., Eastmond D.A. (2006). Tetraploidy and chromosomal instability are early events during cervical carcinogenesis. Carcinogenesis.

[74] Galipeau P.C., Cowan D.S., Sanchez C.A., Barrett M.T., Emond M.J., Levine D.S., Rabinovitch P.S., Reid B.J. (1996). 17p (p53) allelic losses, 4N (G2/tetraploid) populations, and progression to aneuploidy in Barrett’s esophagus. Proc. Natl. Acad. Sci. USA.

[75] Davoli T., de Lange T. (2011). The causes and consequences of polyploidy in normal development and cancer. Annu. Rev. Cell Dev. Biol..

[76] Dewhurst S.M., McGranahan N., Burrell R.A., Rowan A.J., Gronroos E., Endesfelder D., Joshi T., Mouradov D., Gibbs P., Ward R.L. (2014). Tolerance of Whole-Genome Doubling Propagates Chromosomal Instability and Accelerates Cancer Genome Evolution. Cancer Discov..

[77] Gao Q., Hauser S.H., Liu X.L., Wazer D.E., Madoc-Jones H., Band V. (1996). Mutant p53-induced immortalization of primary human mammary epithelial cells. Cancer Res..

[78] Gollahon L.S., Shay J.W. (1996). Immortalization of human mammary epithelial cells transfected with mutant p53 (273his). Oncogene.

[79] Stampfer M.R., Garbe J., Nijjar T., Wigington D., Swisshelm K., Yaswen P. (2003). Loss of p53 function accelerates acquisition of telomerase activity in indefinite lifespan human mammary epithelial cell lines. Oncogene.

[80] Herr A.J., Ogawa M., Lawrence N.A., Williams L.N., Eggington J.M., Singh M., Smith R.A., Preston B.D. (2011). Mutator suppression and escape from replication error-induced extinction in yeast. PLoS Genet..

[81] Sniegowski P.D., Gerrish P.J., Johnson T., Shaver A. (2000). The evolution of mutation rates: Separating causes from consequences. Bioessays.

[82] Nowak M., Schuster P. (1989). Error thresholds of replication in finite populations mutation frequencies and the onset of Muller’s ratchet. J. Theor. Biol..

[83] Andor N., Maley C.C., Ji H.P. (2017). Genomic Instability in Cancer: Teetering on the Limit of Tolerance. Cancer Res..

[84] Zhang H., Jin Y., Chen X., Jin C., Law S., Tsao S.W., Kwong Y.L. (2006). Cytogenetic aberrations in immortalization of esophageal epithelial cells. Cancer Genet. Cytogenet..

[85] Jin Y., Zhang H., Tsao S.W., Jin C., Lv M., Strömbeck B., Wiegant J., Wan T.S., Yuen P.W., Kwong Y.L. (2004). Cytogenetic and molecular genetic characterization of immortalized human ovarian surface epithelial cell lines: Consistent loss of chromosome 13 and amplification of chromosome 20. Gynecol. Oncol..

[86] Coursen J.D., Bennett W.P., Gollahon L., Shay J.W., Harris C.C. (1997). Genomic instability and telomerase activity in human bronchial epithelial cells during immortalization by human papillomavirus-16 E6 and E7 genes. Exp. Cell Res..

[87] Savelieva E., Belair C.D., Newton M.A., DeVries S., Gray J.W., Waldman F., Reznikoff C.A. (1997). 20q gain associates with immortalization: 20q13.2 amplification correlates with genome instability in human papillomavirus 16 E7 transformed human uroepithelial cells. Oncogene.

[88] Liu S., Hatton M.P., Khandelwal P., Sullivan D.A. (2010). Culture, immortalization, and characterization of human meibomian gland epithelial cells. Investig. Ophthalmol. Vis. Sci..

[89] Garbe J.C., Bhattacharya S., Merchant B., Bassett E., Swisshelm K., Feiler H.S., Wyrobek A.J., Stampfer M.R. (2009). Molecular distinctions between stasis and telomere attrition senescence barriers shown by long-term culture of normal human mammary epithelial cells. Cancer Res..

