# Supplementary material for: Generation of Immortalised But Unstable Cells after hTERT Introduction in Telomere-Compromised and p53-Deficient vHMECs
Source: Int J Mol Sci. 2018 Jul 17;19(7):2078. doi: 10.3390/ijms19072078 (PMC6073565; doi:10.3390/ijms19072078)
Supplement: Supplementary file 1 [file ijms-19-02078-s001.pdf]

Supplementary Materials

Table S1. Chromosome analysis of young vHMECs at PD22.

**Karyotype**	**number**
46, XX	15
46, XX, ctb(13q)	1
45, XX, fus(11p;22q)	1
43, XX, ctb(6q), -3, -16, -22	1
46, XX, ctb(10q)	1
46, XX, fus(21p;19q)	1
46, XX, nrt(14p)	1
46, XX, +ace	1
45, XX, fus(2q;17q)	1
46, XX, nrt(4p), fus(19q;22q)	1
92, XXXX, fus(3p;3q)	1
94,XXXX, +2, +12, fus(19q;20p), -20, +22	1
**TOTAL**	**26**

Table S2. Chromosome analysis of aged vHMECs at PD32.

Karyotype	number
43, X, nrt(5q), fus(9q;22q), fus(19p;22q)	1
45, XX, fus(Dq;22q), +csb	1
45, XX, fus(12q;13q)	1
45, X, nrt(1q), fus(14q;22p), csb(Xp)	1
43, XX, fus(12q;14q), fus(14q;22p), -20	1
43, XX, dic(9q;?;14q), -18, -22	1
45, XX, fus(9q;12q), nrt(22q,14q)	1
45, XX, fus(12q;?;14q)	1
46, XX, +csb, +csb	1
44, XX, +csb	1
45, XX, fus(14p;22q)	1
43, XX, fus(2q;14q)	1
42, XX, fus(20q;22q), several losses	1
44, X, fus(12q;?;22q), -X	1
45, XX, fus(12q;16), -21, +mar	1
44, XX, i(14q), +14	1
43, XX, fus(9q;14q), nrt(22q;14q), fus(18p;21p)	1
?, XX, fus(2q;14q)	1
92,XXXX, fus(9q;21q), fus(9q;21q)	1
92,XXXX, fus(9q;21q), fus(14p;14p)	1
**TOTAL**	**20**

Table S3. Chromosome analysis of vHMEC-shp53 at PD29.

**Karyotype**	**number**
23, mar(Xq), dic(2q;?;2q), dic(2q;?;3q), dic(12q;18q), +11	1
35, XX, dic(2q;17q), dic(3q;5q), dic(8q;20q), ntr(9q;?), +12, +12, ntr(12q;?), dic(14q;21q+), +ace, +ace, +ace	1
Pseudotriploid, dic(7p;18q), +ace	1
43, XX, ntr(3q;?), -1, -7, -17	1
76, XXXX, dic(3q;21q), del(5p), dic(18q;21p)	1
40, XX, dic(12q;22q), csb(12p), +ace, +ace	1
40, XX, dic(9q;11p)	1
29, X, dic(2q;16q), dic(3q;17q), dic(12p;?), csb(?)	1
45, XX, t(4q;9q), nrt(11p;?), -14	1
37, XX, +mar	1
42, XX, tric(7p;?;15q), dic(21q;19q), -16	1
41, X, tric(12q;21;?;10p), dic(19q;20p), +mar	1
33, XX, dic(3q;12q), tric(19q;?;14q), +ace, +mar	1
31, XX, +csb	1
44, XX, dic(14q;20p), dic(15p;17q)	1
42, XX, dic(11p;?), tric(21q;19;22q), +ace	1
40, XX, dic(2p;17p), dic(12p;16q), -5, -9, -14, -16	1
80, del(2p), nrt(2q;?), tric(3q;17;?;22q), dic(7q;?;15p), del(14p), dic(17q;?;22q), dic(20p;21p)	1
39, XX, dic(19p;21q)	1
40, X, dic(2p;?;12p), nrt(3p;?), dic(4q;7p), dic(10p;?), nrt(16q;?), tetrac(17q;22;X;20p), +ace, +csb	1
Pseudotriploid, r(3), r(3), tric(12q;?;20q), tric(12q;?;20q), dic(21q;19), dic(22q;?)	1
43, XX, tric(2q;17;12q), -16	1
38, XX, dic(12q;12q), dic(21q;?;19p), dic(16q;?)	1
**TOTAL**	**23**

Table S4. Chromosome analysis of vHMEC-hTERT at PD76.

**Karyotype**	**number**
46, XX	4
47, XX, +20	34
47, XX, nrt(15q;?), +20	1
47, XX, nrt(5q;?), +20	1
47, XX, del(17p), +20	1
47, XX, del(10p), +20	2
46, XX, del(18p)	1
46, XX, nrt(16q;?)	1
46, XX, -16, +mar	1
**TOTAL**	**46**

Table S5. Chromosome analysis of vHMEC-shp53-hTERT at PD47.

**Karyotype**	**number**
46, XX	16
45, XX, -14	1
45, XX, -22	1
46, XX, -11, +mar	1
46, XX, -20, +mar	1
41, X, -7, -12, -17, -18	1
45, XX, tric(9p;?;15p), nrt(12p:?), -19, nrt(20p;?)	1
46, XX, nrt(21q;?)	3
46, XX, dic(20q;?;17p)	1
46, XX, nrt(12p:?), dic(18p;19p), nrt (21q;?)	1
46, XX, dic(9p;?;21p), nrt(12p:?)	1
45, XX, nrt(12p:?), -19	1
46, XX, nrt(12p:?)	1
46, XX, nrt(9p;?), nrt(12p:?), dic(21p;?)	1
46, XX, nrt(9p;?), nrt(12p;?), nrt(20p;?)	1
46, XX, nrt(9p;?), nrt(12p;?), nrt(21p;?)	1
48, XX, nrt(12p;?), nrt(21q;?), +del, +ace, +ace	1
45, XX, del(9p), nrt(12p;?), -16, +ace	1
45, XX, idic(7p;7p), +7, dic(20p;?), tric(22q, ?; ?), -21	1
45, XX, nrt(12p:?), -21	6
46, XX, dic(6p;14p), dic(9p;?), nrt(12p;?), dic(13p;14p), nrt(21q;?)	1
46, XX, nrt(3q;?), del(Xq)	1
46, XX, nrt(3q;?)	2
47, XX, nrt(9p;?), nrt(21p;?), +21	1
45, XX, del(3p), -21	1
46, XX, dic(20q;?)	1
46, XX, del(1q), nrt(17q;?)	1
46, XX, nrt(9p;?), nrt(12p;?), nrt(21p;?)	1
46, XX, del(3p), dic(20q;?)	1
92, XXXX	1
87, XXXX, -12, nrt(12p:?), nrt(12p:?), -21, -22	1
**TOTAL**	**54**

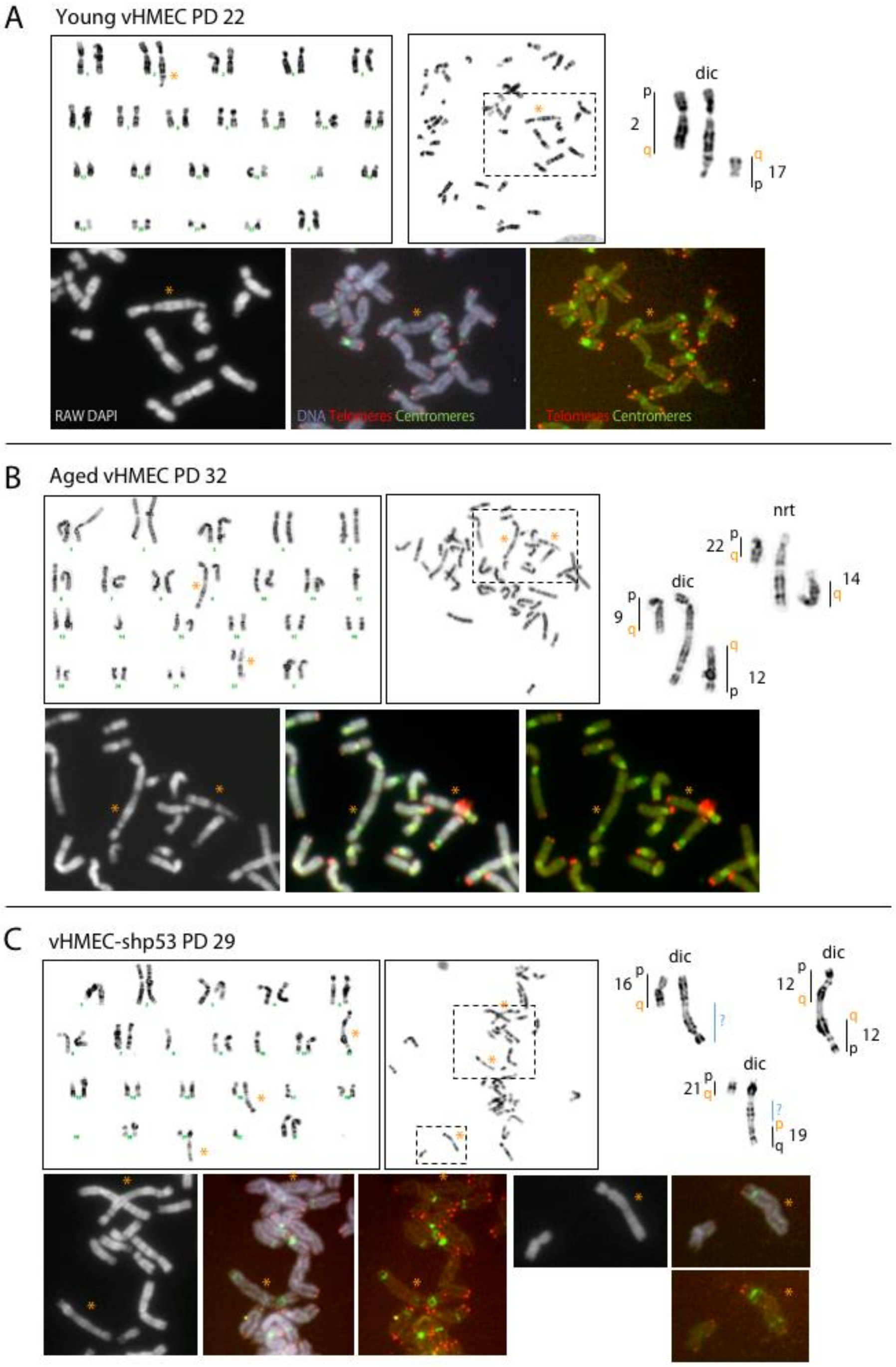

Figure S1. Procedures of the cytogenetic analysis of the finite cell lines. The karyotype, the metaphase spread and the reorganised chromosomes are shown. The rearranged chromosomes are indicated with an asterisk. Moreover, partial images from the metaphase after in situ hybridisation with pancentromeric and pantelomeric PNA probes, which corresponds to the discontinuous line box, are shown. From left to right, the raw image of DAPI; the merged image of DNA (blue) and PNA centromeric (green) and telomeric (red) probes; and the combination of PNA probes only. (A) Karyotype of a young vHMEC at PD22 showing an end-to-end fusion between 2q and 17q. (B) Karyotype of an aged vHMEC at PD32 showing an end-to-end fusion between 9q and 12q as well as a non-reciprocal translocation (nrt) between 22q and 14q. (C) Karyotype of a vHMEC-shp53 at PD29 showing an end-to-end fusion between 12q and 12q as well as two dicentric chromosomes not completely defined. Note the absence of telomere FISH signals at the fusion point of all the dicentric chromosomes displayed.

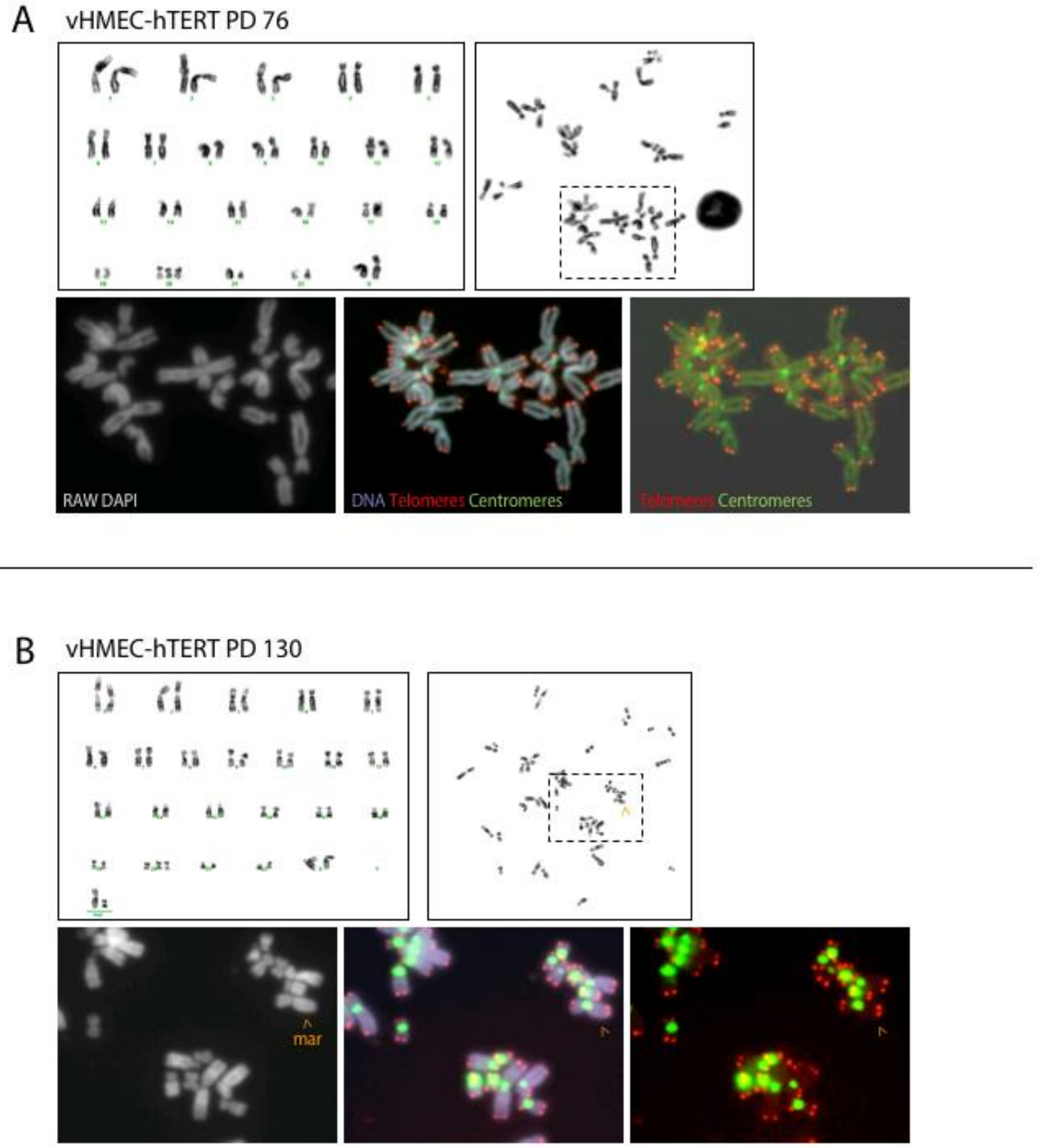

Figure S2. Procedures of the cytogenetic analysis of the vHMEC-hTERT cell line. The karyotype and the metaphase spread are shown. Moreover, partial images from the metaphase after *in situ* hybridisation with pancentromeric and pantelomeric PNA probes, which corresponds to the discontinuous line box, are shown. From left to right, the raw image of DAPI; the merged image of DNA (blue) and PNA centromeric (green) and telomeric (red) probes; and the combination of PNA probes only. **(A)** Karyotype of vHMEC-hTERT at PD76 showing trisomy 20. **(B)** Karyotype of vHMEC-hTERT at PD130 showing two marker (mar) chromosomes in addition to trisomy 20. Open arrows indicate one of the marker chromosomes.

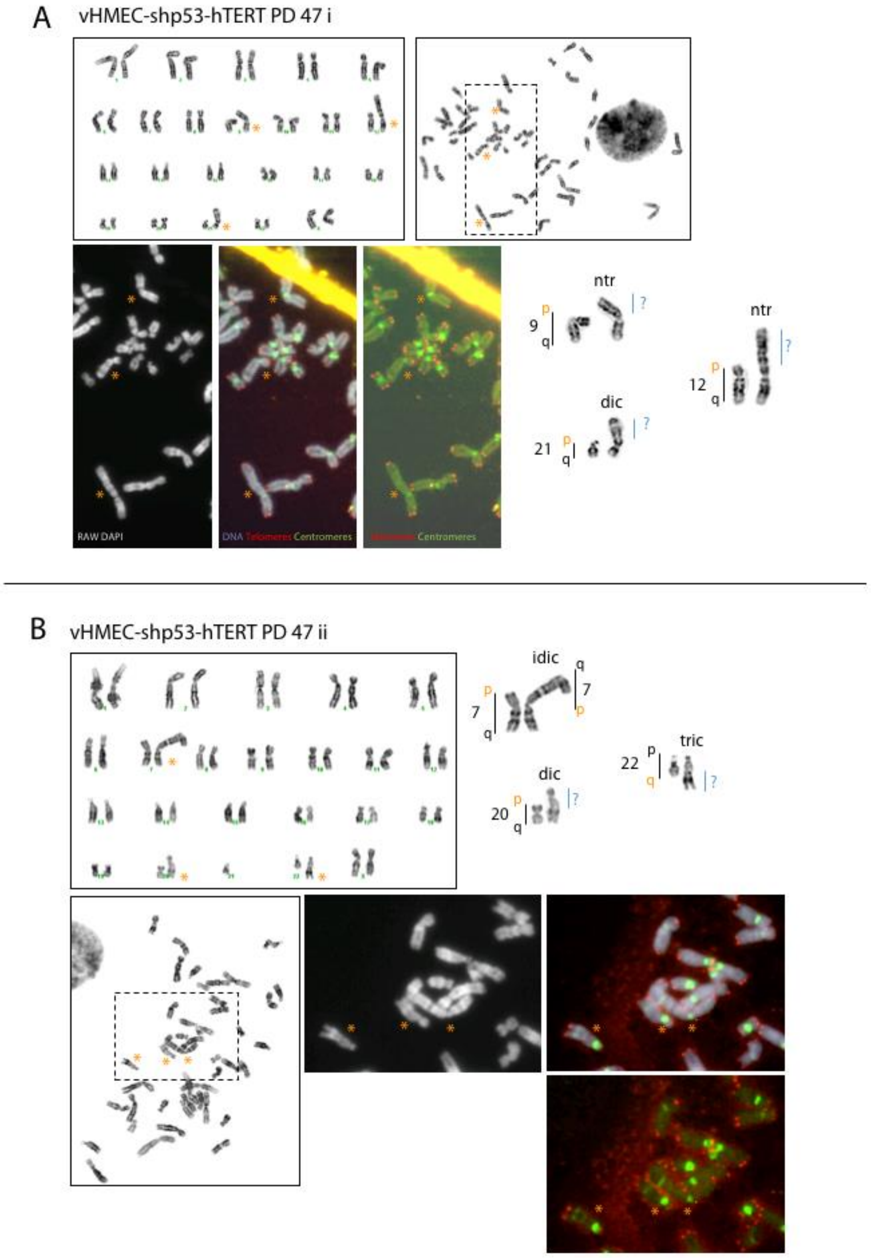

Figure S3. Procedures of the cytogenetic analysis of the vHMEc-shp53-hTERT cell line. The karyotype, the metaphase spread and the reorganised chromosomes are shown. The rearranged chromosomes are indicated with an asterisk. Moreover, partial images from the metaphase after *in situ* hybridisation with pancentromeric and pantelomeric PNA probes, which corresponds to the discontinuous line box, are shown. From left to right, the raw image of DAPI; the merged image of DNA (blue) and PNA centromeric (green) and telomeric (red) probes; and the combination of PNA probes only. **(A)** Karyotype of a vHMEC-shp53-hTERT at PD47 showing two ntr, as well as one dicentric chromosomes not completely defined. **(B)** A second karyotype of a vHMEC-shp53-hTERT at PD47 showing an end-to-end fusion between 7p arms of two distinct chromosomes, as well as one dicentric and one tricentric chromosomes not completely defined. Note the absence of telomere FISH signals at the fusion point of all the dicentric chromosomes displayed.
